# Temporal and Spatial Distribution of Mercury in Gulls Eggs from the Iberian Peninsula

**DOI:** 10.1007/s00244-018-0584-0

**Published:** 2018-12-18

**Authors:** M. Glória Pereira, Alan Lawlor, Albert Bertolero, Sergi Díez, Richard F. Shore, Silvia Lacorte

**Affiliations:** 1 Centre for Ecology and Hydrology, Lancaster Environment Centre, Library Avenue, Bailrigg, Lancaster, LA1 4AP UK; 2 Associació Ornitològica Picampall de les Terres de l’Ebre, Amposta, Catalonia Spain; 3 Department of Environmental Chemistry, Institute of Environmental Assessment and Water Research, IDÆA-CSIC, Jordi Girona 18-26, 08034 Barcelona, Catalonia Spain

## Abstract

We examined how coastal mercury contamination varied spatially and temporally across the Iberian Peninsula by measuring mercury concentrations in the eggs of the sentinel biomonitor yellow-legged gull (*Larus michahellis*). Samples were collected from eight colonies that ranged from the Atlantic across the south and northern areas of the Mediterranean. We also measured Hg residues in eggs of the one of the most endangered gull species in the world, the Audouin’s gull (*Larus audouinii*) from the Ebro Delta, where colonies of yellow-legged and Audouin’s gull co-occur. Fresh eggs were collected in 2009 and 2016 and samples were pooled from each colony for analysis. Mercury concentrations in yellow-legged gulls ranged between 0.4 and 2.8 mg/kg dry weight (dw); although there were no significant differences in concentrations between sampling periods, significant differences were found between colonies. Higher concentrations were associated with northern Mediterranean colonies (Columbretes and Ebro Delta), likely due to proximity to emission sources, circulatory marine currents and diet composition. Mercury concentrations in yellow-legged gull eggs were lower than those reported to result in impaired hatching. Residues in Audouin’s gull eggs from the Ebro Delta were significantly higher (4.0–5.6 mg/kg dw) than those in yellow-legged gull from the same location, probably associated with dietary differences. Mercury levels in Audouin’s gull were ten times above the benchmark suggested to reduce nest success by 10%. Overall, these results raise concern for adverse health impacts in this protected seabird species and further investigation in Audouin’s gull eggs from the Ebro Delta is recommended.

Mercury (Hg) is a toxic, nonessential heavy metal that is released into the environment from natural and anthropogenic sources. Its wide spatial distribution is linked to emissions from various sources, such as fossil fuel combustion, chlor-alkali plants, and agriculture, and is considered a global environmental pollutant (Driscoll et al. 2013).

In Spain, point-source emissions of Hg include eight chlor-alkali plants and Hg mining activity responsible for most contamination at local and regional scales. Even though Hg mining ceased in 2004, Hg is still detected in a wide range of environmental matrices, e.g., in soils and plants from the mining sites in Almaden (Molina et al. 2006) and from an abandoned cinnabar mining site (Garcia-Sanchez et al. 2009); in food web components collected close to a chlor-alkali plant (Carrasco et al. 2008, 2011a); in downstream (Carrasco et al. 2011b) river water and sediments of Catalonia (Roig et al. 2011) and Asturias (Garcia-Ordiales et al. 2018); and in marine macrophytes and sediments of the Spanish Mediterranean coast (Sanchiz et al. 2000).

Due to its physical and chemical properties, Hg occurs in the environment in different forms and it is of particular concern in aquatic ecosystems. Here, inorganic Hg is biotransformed into the highly toxic, methylmercury (MeHg), which tends to bioaccumulate and bioconcentrate in food webs. Although MeHg tends to represent less than 1% of the total Hg in fresh and marine waters, c. 96% of the total Hg in top predators is MeHg (Wiener et al. 2003). Hg persistence is also attributed to its slow metabolism by vertebrates (Walker and Livingstone 1992). High levels of Hg exposure have been related to a wide range of adverse effects (Giesy et al. 1994), including reprotoxicity (Furness 1993; Grassman et al. 1998; Boening 2000; Kenow et al. 2003), with MeHg being considered highly neurotoxic for humans and wildlife (Diez 2009).

Organisms that readily respond to contamination events can be used as bioindicators of contamination, providing information on spatial and temporal variation in environmental concentrations (Bishop et al. 1995). Birds and their eggs have been used as such indicators with the use of coastal-nesting birds as sentinels to detect changes in the bioavailability and transfer of Hg through coastal marine systems being well established (Pereira et al. 2009; Dittmann et al. 2012; Champoux and Boily 2017). Hg residues, in particular, have been measured in eggs (contents rather than in eggshells, Peterson et al. 2017), because they tend to have a consistent composition unlike body tissues, which can vary markedly between and within species in mass and lipid content. Furthermore, Hg accumulation in eggs of income breeders species is attributed to the feeding habits of the female in the breeding area (Sanpera et al. 2000), reflecting recent dietary exposure (1–2 weeks before egg laying) (Furness 1993; Becker et al. 1994). Therefore, eggs appear to be good indicators of localised and contemporary environmental exposure. In Spain, Hg concentrations have been reported in the eggs of common terns (*Sterna hirundo)* (Guitart et al. 2003) and of Audouin’s gulls (*Larus audouinii*) from the Ebro Delta (Morera et al. 1997; Garcia-Tarrason et al. 2013), in Andouin’s gull eggs from Chafarinas Islands (Sanpera et al. 2000), and in yellow-legged gull (*Larus michahellis*) eggs from the Medes Islands (Sanpera et al. 1997).

Studies on Hg residues in gull eggs conducted to date in Portugal and Spain have focused mostly on single locations and do not provide information on how contamination patterns vary spatially. The Iberian Peninsula has both Mediterranean and Atlantic coasts and pollution levels are expected to vary as a result. The Mediterranean is an enclosed basin with limited exchange of deep water with the outer oceans; water circulation is dominated by salinity and temperature differences rather than winds. These geographic and climatological conditions make the Mediterranean a sink for pollutants from river and treated and untreated wastewater discharges, and run-off (Sanchez-Avila et al. 2009). Furthermore, there are areas that contain long-established chemical industries and associated contamination; for instance, large amounts of industrial waste containing high Hg concentrations (up to 436 µg/g) were dumped into a reservoir adjacent to the Ebro river (Carrasco et al. 2008, 2011a) with potential transfer downstream to the delta (Carrasco et al. 2011a, b). Compared with the Mediterranean coast, pollution in the Atlantic coast of the Iberian Peninsula is lower due to dilution by the seawater and oceanic currents (Yamashita et al. 2005), although there are hotspots of contamination associated with human activities (Rubio et al. 2000).

Selenium (Se) often co-occurs with Hg, and there is some evidence that Se may be protective of Hg toxicity in several organisms (Ralston and Raymond 2013; Cusack et al. 2017; Spiller 2018). We also determined Se concentrations in the eggs collected in 2009, because whilst Se is an essential element for developing embryos, excess levels produce embryotoxic and teratogenic effects, embryonic malformations, and increased mortality. Also, simultaneously high concentrations of Se and Hg can have synergistically negative effects on embryonic development in birds (Heinz and Hoffman 1998).

The overarching goal of the present study was to investigate how concentrations of Hg vary across the Iberian Peninsula, both in the Atlantic and Mediterranean coasts. Such information is important to understand which areas, and the wildlife that they support, may be at most risk from contamination. We assessed this variation by measuring Hg in the eggs of the bio-indicator species, yellow-legged gull (*Larus michahellis*). We used this species because of its wide breeding distribution and because it is a coastal feeder (Ramos et al. 2009). We report temporal and spatial variations in total Hg concentrations in eggs taken from nesting colonies around the Iberian Peninsula in 2009 and 2016. Where possible, we compare Hg concentrations in yellow-legged gull eggs with those in the eggs of Audouin’s gulls, a highly endangered species. Until the mid-2000s, the Ebro Delta hosted 70% of the world’s breeding population (Fernandez-Chacon et al. 2013), but this has declined to only 3% in 2017 (Genovart et al. 2018). Our goal was to determine whether residues in yellow-legged gull eggs can be used as a surrogate measure of exposure in Audouin’s gulls. Finally, and given the uncertainty with regard to the protective effect of Se on Hg toxicity, we relate Hg to Se concentrations.

## Materials and Methods

### Sampling Sites and Egg Collection

**Fig. 1:**
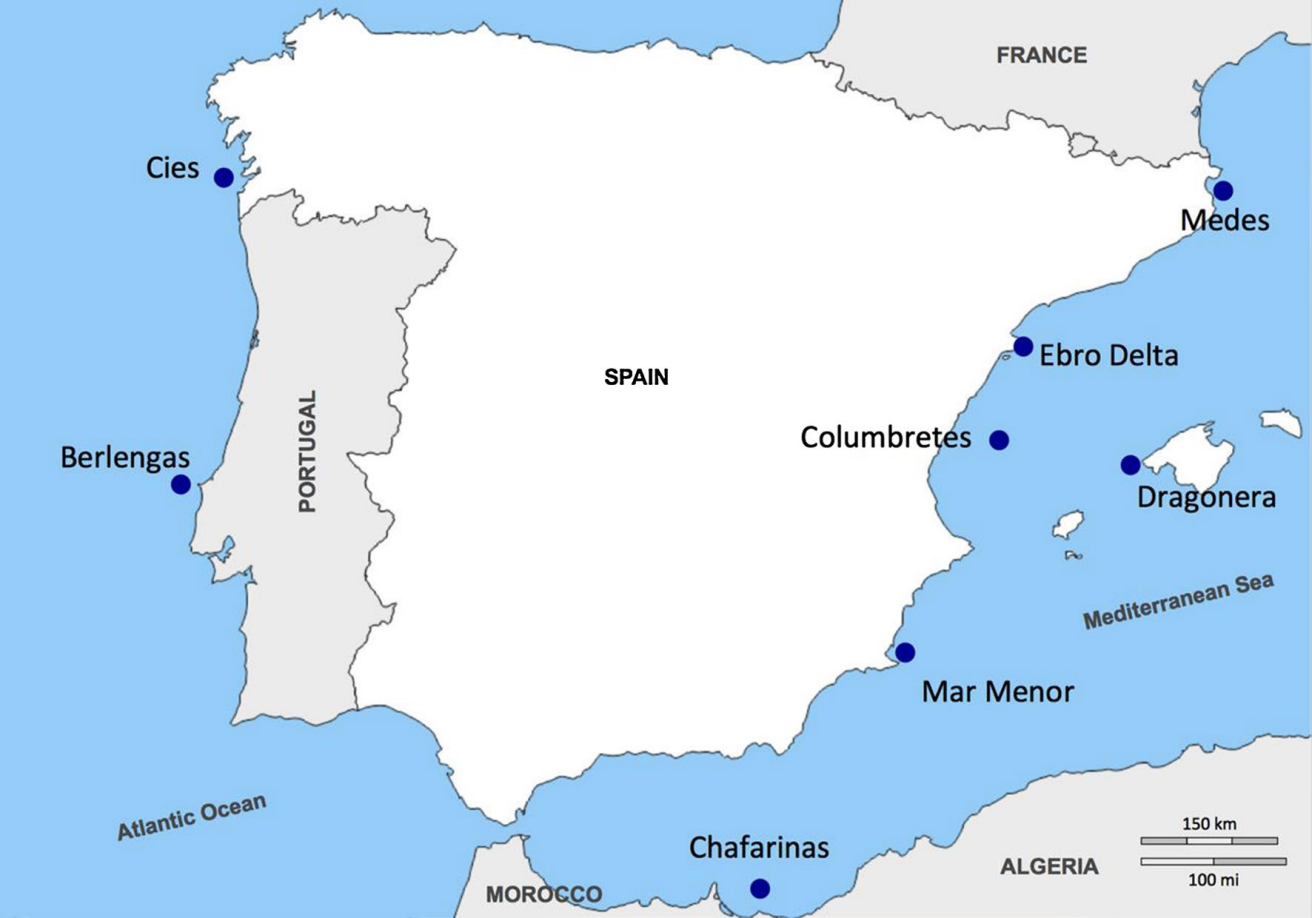
Colony locations from which eggs were sampled. These areas represent the most important gull colonies of the Iberian Peninsula and they all are declared Special Protection Area for birds

Fresh, yellow-legged gull eggs were collected from eight colonies, in Spring 2009 and 2016, with the permission of National and Natural Parks. The sampling areas were representative of two geographical areas: the Atlantic Ocean and the Mediterranean Sea. The Atlantic colonies were Berlengas and Cies Islands, with the Mediterranean colonies located at the northern (NM: Medes, Ebro Delta and Columbretes Islands) and southern Spanish coasts (SM: Dragonera, Mar Menor and Chafarinas Islands) (Fig. 1; Table 1). The NM and SM sites are influenced by the Northern current and the Algerian current, respectively (Millot 1987, 1999). The characteristics of the different sites, including distance from mainland and anthropogenic pressures, are described in Table 1. Fresh Audouin’s gull eggs also were collected from the Ebro Delta in the 2009 and 2016 breeding seasons.

**Table 1:** Description of the colonies sampled in 2009 and 2016, ordered from northeast to northwest of the Iberian Peninsula, area of the colony, *L. michahellis* population in all colonies and *L audouin* population in the Ebro Delta, food resources, distance from main human settlements and human impact

Colony	Area (km^2^)	*L. michahellis* population	Resources	Distance from human settlements (km)	Human impact	References
Medes 3°13′E 42°02′N	1.8	7291	Refuse tips	0.87	Industry, tourism	Bosch et al. (1994)
Ebro Delta 0°40′E 40°35′N	320	9744 14,177 *L. audouin*	Refuse tips, waste, crabfish Benthic-mesopelagic fish	7.5	Rice fields, chlor-alkali plant	Ramos et al. (2009) and Oro et al. (2009)
Columbretes 0°41′E 39°54′N	0.19	500	Pelagic fish	53.78	Tourism, agriculture	Ramos et al. (2009)
Dragonera 2°18′E 39°35′N	3	4500	Refuse tips	0.94	Textile, leather, tourism	Bermejo et al. (2009)
Mar Menor 0°42′W 37°43′N	0.165	1272	Refuse tips, brackish, freshwater and pelagic preys	1.57	Agriculture, greenhouses	Ramos et al. (2009)
Chafarinas 2°25′W 35°10′N	0.525	5700	Epipelagic fish	6.67	Agriculture	Arcos et al. (2002) and Pedrocchi et al. (2002)
Berlengas 9°30′E 39°24′N	0.788	23,000	Refuse tips	10.32	Industry, agriculture, boating	Moreno et al. (2010)
Cies 8°54′W 42°13′N	4.4	15,654	Most pelagic preys, but also benthic preys and refuse tips	2.54	Fishing, boating, chlor-alkali	Moreno et al. (2010)

At each colony, 36 eggs were randomly collected from 3 different areas within the colony (hereafter termed subcolonies) such that 12 eggs were taken per subcolony. We adopted the sampling method described by Vicente et al. (2012), who reported that sample pooling successfully encompassed within-colony variability of residue concentrations. It also followed the recommendations of the United Nations Environment Programme and OSPAR for sampling and processing of biota samples (OSPAR Commission 2007; UN Environment). We collected the first layed egg, because it typically contains the highest within-clutch contaminant concentration, resulting from transfer from female to eggs (Sanpera et al. 1997). The contents of 12 eggs from each subcolony were pooled. Thus, there were three pooled samples per colony for analysis, except where limited colony size meant that only 12 eggs in total were collected (Columbretes in both years, Mar Menor and Berlengas in 2016 and Dragonera in 2009). In those cases, there was only one pooled sample per colony for analysis. No eggs were collected from Dragonera in 2016.

### Hg Analysis

Hg concentrations were measured in approximately 1-g subsamples that were dried to constant weight at 80 °C for 24 h, solubilised at room temperature overnight in 2 ml of (Analar) nitric acid, then heated at 90 °C (20 min) and then 120 °C (1 h). To aid the digestion of the organic matter, 0.5 ml of 30% hydrogen peroxide was added, which was then heated at 120 °C for 15 min. Digests were diluted with double-deionised water to a known volume and 10% acid strength. The resulting digests were analysed for Hg by Inductively Coupled Plasma—Mass Spectrometry (ICP-MS) using a Perkin Elmer DRCII ICP-MS with standard operating conditions. Hg concentrations are expressed as mg/kg dry weight (dw). For quality control, blanks and three certified reference materials (pig kidney, TORT-2 and DOLT-4) were run alongside samples; average recoveries (*n* = 3) in the reference materials were 93% (RSD = 2.1%), 104% (RSD = 10.1%), and 98.6% (RSD = 17.3%), respectively. The sample limit of detection (LoD) and was determined as 3 × the standard deviation of the blank and was 0.1 mg/kg. Samples were blank corrected, when blank concentrations were above LoD.

At the same time that we analysed Hg in the 2009 eggs, a suite of metals also was analysed (data not presented here), including selenium (Se). The average recoveries (*n* = 3) for Se in the reference materials (pig kidney, TORT-2 and DOLT-4) were 99% (RSD = 5.1%), 131% (RSD = 7.4%), and 111% (RSD = 3.3%) and the LOD was 0.05 mg/kg. The molar ratio of Hg to Se was calculated by dividing the calculated number of moles of Hg with those of selenium.

### Statistical Analysis

Hg concentrations in yellow-legged gulls from the various locations and for both sampling dates were log10 transformed for analysis, and so geometric means and ± geometric 95% confidence intervals (CI) are presented. The significance of differences between years and sites were analysed using a general linear model that included sampling year and site as random variables; differences between sites were assessed using Tukey post hoc tests. Data for Hg concentrations for yellow-legged gulls from Dragonera were not included in the analysis, because no eggs were sampled in 2016. The underlying assumptions of the model of homogeneity of variance (Barlett’s test) and normality of residuals (Kolmogorov–Smirnov test) were met. A pairwise *t* test was used to compare Hg concentrations between Audouin’s and yellow-legged gull eggs from the Ebro Delta.

## Results and Discussion

### Temporal Variation in Hg in Yellow-Legged Gulls

**Fig. 2:**
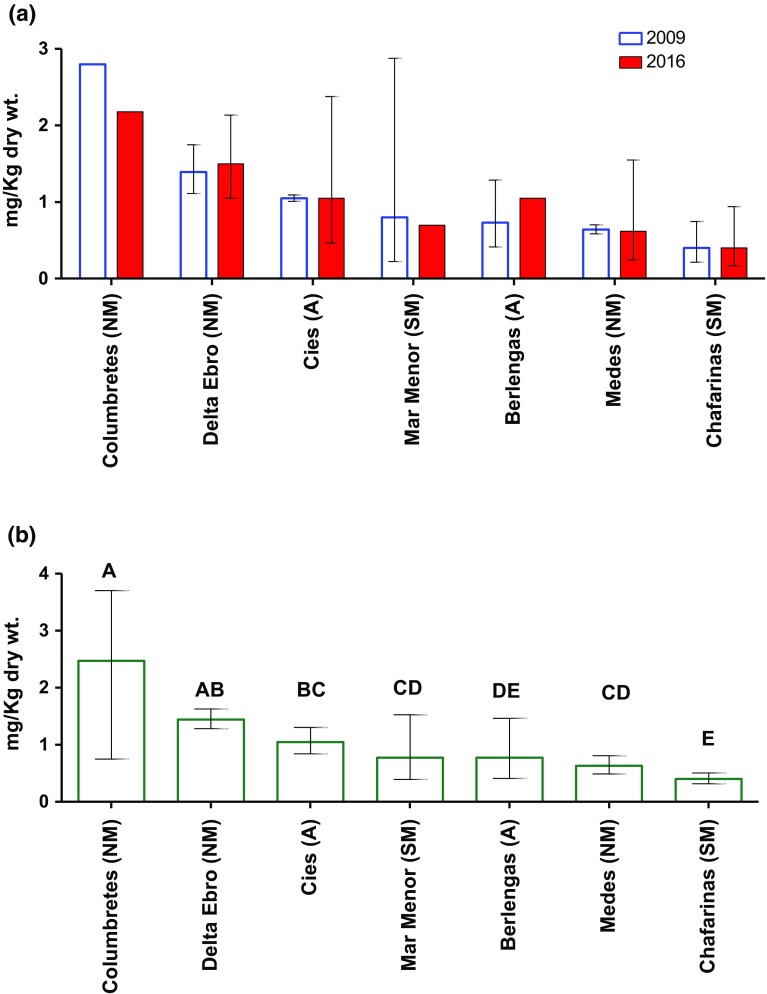
Geometric mean (± 95% CI) concentration of total Hg in yellow-legged gull eggs collected from various colonies on the Iberian Peninsula. **a** Hg concentrations (mg/kg dw) in eggs collected in 2009 and 2016. **b** Data from 2009 and 2016 combined. Letters on top of bars indicates significant differences

The results of the Hg analyses in eggs collected from the various sites in 2009 and 2016 are presented in Fig. 2a. There was no significant difference in concentrations between the 2 years across the sites (*F*_(1,26)_ = 0.02, *P* = 0.876). This suggests that contamination remained relatively unchanged over the 7-year period. When colonies were grouped as Atlantic, NM, or SM, there were, again, no obvious differences in temporal trends, with the  % change between 2009 and 2016 ranging between − 0.1 and 44%, − 22 and 7.5%, and − 0.1 and − 12.9%, respectively (Fig. 2a). The largest % changes were noticed in colonies with only one pooled sample in one or both sampling years; thus, in these cases data may be considered less robust. Changes in geometric mean concentration for sites with multiple pooled samples in either sampling year were only from − 3.6 to 7.5%. At the Ebro Delta, the removal of upstream contaminated sediments began in 2013 (Garcia-Tarrason et al. 2013). However, no marked changes in Hg concentrations in yellow-legged gull eggs (Fig. 2a) seem to be associated with dredging operations, perhaps reflecting the generalist diet of this species.

Overall, the lack of temporal changes in concentrations across the Iberian Peninsula contrasts with studies of Hg contamination in bird eggs elsewhere. Hg concentrations decreased in herring gull (*Larus argentatus*) eggs in the Great Lakes between 1970 and 2000, but temporal trends have since varied between locations, with increases in some cases and further declines in others (Blukacz-Richards et al. 2017). Braune et al. (2016) observed that residues in glaucous gull (*Larus hyperboreus*) eggs in the Canadian Artic decreased between 1993 and 2003 but subsequently started to increase, although similar temporal trends were not always observed in other species measured in the same study. Mercury concentrations also have been shown to have increased since the year 2000 in common loons (*Gavia immer*; Meyer et al. 2011) and in bald eagles (*Haliaeetus leucocephalus*; Pittman et al. 2011) in North America.

### Spatial Variation in Hg Concentrations in Yellow-Legged Gull Eggs

Hg concentrations in yellow-legged gull eggs ranged between 0.4 and 1.77 mg/kg dw, except in Columbretes, which contained up to 2.8 mg/kg dw. There were significant differences in concentrations between sites (*F*_6,26_ = 1877, *P* < 0001; Fig. 2b). They were highest in two of the three NM sites (i.e., Columbretes and Ebro Delta) and were significantly higher than in the two SM sites (Mar Menor and Chafarina; Fig. 2b). Concentrations in the single 2009 pooled sample from Dragonera (not included in the statistical analysis) was 0.9 mg/kg dw, which is 1.5 and 3 times lower than the 2009 concentrations in the Ebro Delta and Columbretes Islands, respectively. Concentrations in Medes were significantly lower than those at the other two northern sites and were similar to those found in the more southern colonies (Fig. 2b) and also to those recorded in 1992 (0.52 ± 0.29 µg/g dw) at the same location (Sanpera et al. 1997). Although there is some evidence that Atlantic fauna accumulate less Hg compared with biota from the Mediterranean (Renzoni et al. 1998), the mean Hg residues in eggs from the two Atlantic colonies generally fell between the values recorded in eggs from the NM and SM colonies (Fig. 2b). The residues obtained in samples from Berlengas were similar to those (1.4 µg/g dw) reported previously for yellow-legged gull eggs from the same area (Monteiro et al. 1999).

The spatial variation of Hg in yellow-legged gull eggs can be explained by the distribution of anthropogenic and natural sources of Hg. The NM Ebro Delta colony is close to industrialised and highly populated areas, and concentrations most likely reflect Hg inputs into the Ebro River from intensive agricultural and industrial activity within its watershed, including inputs from nearby chemical plants (Morera et al. 1997; Sanchiz et al. 2000; Sanpera et al. 2000). However, the highest mean Hg concentration was found in eggs from Columbretes Islands (Fig. 2b). Compared with the Ebro Delta, the Columbretes Islands are more isolated from major point sources of Hg, and so anthropogenic inputs alone cannot account for the relatively high concentrations at this colony, up to twofold higher than in the Ebro Delta. The Catalan-Balearic Sea in the NM carries cold northern waters from the Gulf of Lion southward along the continental slope in the Balearic Sea, and this current bifurcates at the northern end of the Eivissa Channel (Millot 1999), potentially transporting atmospheric Hg from the North towards Columbretes.

We expect that dietary differences between colonies may also contribute to the intercolony variation of Hg and in future work we plan to perform stable isotopes analyses to ascertain whether diet contributes to the observed differences in mercury concentrations. Yellow-legged gulls in the Iberian Peninsula feed in both marine and freshwater environments, prey on smaller seabirds, and also take the eggs of terrestrial and coastal birds (Ramos et al. 2009). Distance to the mainland is another factor influencing feeding ecology, with scavenging of waste from rubbish tips a more important part of the diet in birds close to the mainland (Ramos et al. 2013). Among the yellow-legged gull colonies studied, Columbretes Islands is the furthest site from the mainland (≈ 54 km; Table 1), and therefore, gulls have a more fish-based rather than waste-based diet (Ramos et al. 2009, 2013), which may account for the higher concentrations measured. Similarly, scavenging on waste tips also may explain why Hg levels in eggs from the Medes Islands were lower than those in eggs from the other NM colonies (Ebro Delta and Columbretes). Yellow-legged gulls from Medes are highly dependent on feeding at refuse tips (Bosch et al. 1994), which may reduce their exposure to Hg compared with feeding on more natural marine or freshwater-based diet (Leonzio et al. 1986).

There was little difference in Hg contamination between the two Atlantic colonies of Cies and Berlengas. The main anthropogenic source of Hg in Cies is the chlor-alkali plant situated on the south coast of the Ria of Pontevedra (Galicia) (Otero and Fernandez-Sanjurjo 2000). Whilst it may be expected that this may contribute to elevated contamination, Cies is an open Atlantic site and currents may dilute contaminant loadings into the food chain. Furthermore, refuse from rubbish tips is an important source in the diet of gulls from both Cies and Berlengas (Moreno et al. 2010). This commonality in diet sources may account for the overall similarity in the Hg levels between the colonies. It was also noticeable that the concentrations in eggs from Cies and Berlengas were similar to those from the (SM) colony at Mar Menor and higher than those from the Chafarinas (Fig. 2b). Given the potential of Atlantic oceanic currents to dilute inputs into the marine food chain, residues in the eggs of Atlantic gulls were expected to be lower than in the SM. However, a study of perfluorinated compounds in yellow-legged gulls eggs from the same colonies found no differences between these two water bodies (Vicente et al. 2012), and, as it has been suggested, temporary influx of Atlantic water into the SM may reduce contamination levels in the southern colonies.

### Comparison Between Hg Concentrations in Audouin’s and Yellow-Legged Gull Eggs

**Fig. 3:**
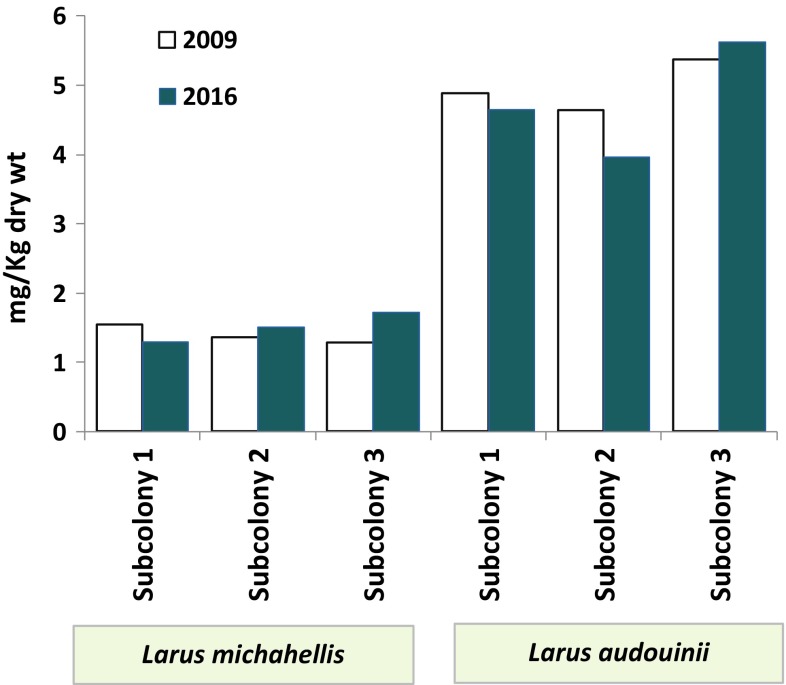
Mercury concentration in the eggs of yellow-legged gulls and Audouin’s gull from three subcolonies of the Ebro Delta collected in 2009 (clear bars) and 2016 (dark bars)

In the three Ebro Delta subcolonies, concentrations were significantly higher for Audouin’s than yellow-legged gulls eggs (3.41 ± 0.52 mg/kg dw (mean ± SD); *t*_(5)_ = − 14.64; *P* < 0.001; Fig. 3). Concentrations were of the same order of magnitude as those reported in other studies in Audouin’s eggs from the Ebro Delta during 1992 (5.05 ± 1.5 mg/kg dw; Morera et al. 1997) and 1994 (5.0 ± 1.54 mg/kg dw; Sanpera et al. 2000), suggesting Hg accumulation has remained fairly unchanged for the past c. 25 years.

The Hg concentrations in Audouin’s gull eggs from the Ebro delta are amongst the highest reported for seabird eggs anywhere (Ackerman et al. 2013). Audouin’s gulls are specialised pelagic marine predators that feed mainly on clupeiform fish (Witt et al. 1981). During the breeding season, they feed preferentially on trawler discards (Oro and Ruiz 1997), which contain both demersal and mesopelagic fish. A continuous release of Hg, from the chlor-alkali plant located 60 km upstream in the dissolve and particulate phase (Turull et al. 2017) would increase the Hg body burden of particular demersal fish species. Hg concentrations in demersal fish from the Ebro Delta can be more than double those in epipelagic fish (Arcos et al. 2002), and consequently Hg concentrations can sharply increase by a diet containing demersal fish (Arcos et al. 2002). Thus, it seems likely that the comparatively high Hg levels in Audouin’s gull eggs around the Ebro delta are a result of higher contamination inputs in this area and to the birds feeding habits. Furthermore, it has been suggested (Henny et al. 2002) that some waterbird species are able to perform mercury demethylation when exposed to high concentrations. This does not appear to be the case with Audouin gulls, although we cannot be certain of this since we did not measure levels in adults.

### Toxicological Implications of Hg in Gull Eggs

Seabirds tend to accumulate high levels of Hg because aquatic conditions facilitate conversion of inorganic Hg into bioavailable MeHg, which bioaccumulates in fish and their predators (Monteiro and Furness 2001; Wiener et al. 2003). In this study, we analysed total Hg, because it has been shown to be a good proxy for the most toxic form of Hg (MeHg) in bird eggs. Ackerman et al. (2013) have reported that, across 22 different species of birds, 96% of the total Hg in eggs is MeHg.

Because the sensitivity of birds to Hg toxicity can differ widely among species (Ohlendorf and Heinz 2011), it is difficult to compare egg Hg concentrations against known toxicity thresholds. Therefore, we have provided several toxicity benchmarks suggested to impair bird reproduction. Laboratory studies revealed that Hg can have deleterious effects in birds including decreased egg weight, embryo malformations, lower hatchability, decreased chick growth, and reduced survival of the young (Thompson 1996; Burger and Gochfeld 1997). For example, a 10% reduction in nest success corresponded with 0.11 mg/kg ww (0.55 mg/kg dw; Jackson et al. 2011) of mercury concentration in the egg. In fact, an egg Hg concentration of 0.6 mg/kg ww, equivalent to approximately 3 mg/kg dw (assuming eggs contain 80% water; Thompson 1996), has been suggested as a conservative generic value below which embryotoxic effects in most avian species are unlikely (Heinz and Hoffman 1998; Shore et al. 2011). Consistent with this, Evers et al. (2003) used Hg concentrations in eggs to estimate reproductive risk in loons (*Gavia immer*) and identified a “moderate risk category” of 2.82–6.10 mg/kg dw that reflected “elevated levels of environmental Hg that may be indicative of significant reproductive impairment in some individuals in some avian species, including loons”. However, the toxicological significance of Hg to seabirds in particular is difficult to interpret (Thompson 1996). An egg Hg concentration > 3.5 mg/kg ww (*c.* 17.5 mg/kg dw) has been suggested as an adverse effect level for common terns (Shore et al. 2011), but Vermeer et al. (1973) found that hatching success of herring gull eggs was unaffected by Hg concentrations in the first-laid egg between 2.3 and 15.8 mg/kg ww (*c.* 10–80 mg/kg dw). In the current study, none of the yellow-legged gull pooled samples from any colony exceeded 3 mg/kg dw. Thus, it seems unlikely that Hg contamination alone currently poses a significant reproductive risk to this species around the Iberian Peninsula. However, Hg concentrations in the Audouin’s gull eggs ranged from 4.0 to 5.9 mg/kg dw. These concentrations are higher than the proposed generic adverse effect concentrations within the range of the “moderate risk” proposed for loons, generally greater than the adverse effect concentration proposed for common terns, but lower than the concentrations associated with no effect in herring gulls, as reported by Vermeer et al. (1973).

Furthermore, in a laboratory study where eggs were injected with methylmercury, it was shown that different bird species, including different species of gulls, have a wide range of sensitivities to this metal (Heinz et al. 2009). For example, based on dose–response curves and the median lethal concentration LC(50), Heinz et al. (2009) found that that the laughing gull (*Larus atricilla*) had relatively low sensitivity (LC(50)s ≥ 1 μg/g Hg), whereas the herring gull (*Larus argentatus*) had medium sensitivity (LC(50)s > 0.25 μg/g Hg but < 1 μg/g Hg).

### Relationship Between Hg and Se

**Fig. 4:**
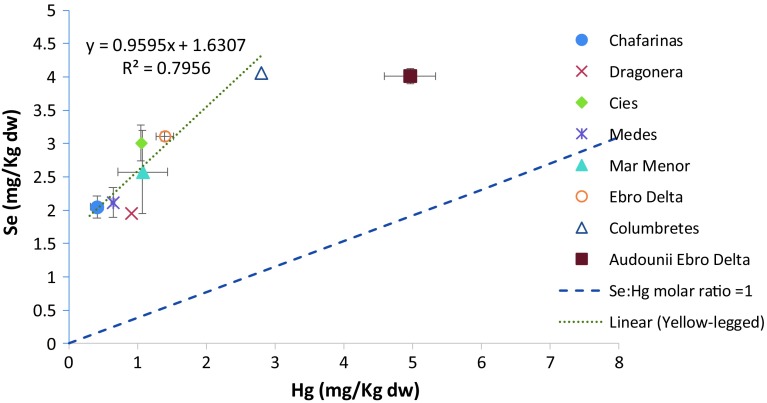
Relationship between mean Hg and Se concentrations in eggs from species collecting in 2009 by sampling sites. The stippled line represents the 1:1 molar ratio between Se and total Hg and the dotted line represents the relationship between Hg and Se for yellow-legged gulls (using all data points)

Selenium concentrations > 5.0 mg/kg dw in eggs are in excess of the normal range, and 6.4 mg/kg dw approaches levels that induce reproductive impairment (Ohlendorf and Heinz 2011). Se concentrations in the present study ranged between 1.9 and 4.1 mg/kg dw, in the yellow-legged gulls and ~ 4 in the Audouin and were below concentrations likely to be associated with adverse effects. However, taken into account all the measurements, there was a significant and positive correlation between yellow-legged egg concentrations of Hg and Se (*r*^2^ = 0.796, *P* < 0.001; Fig. 4). Positive correlations also were found for eggs of the common loon (Scheuhammer et al. 2001), Forster’s tern (*Sterna fosteri*), and black skimmer (*Rynchops niger*; King et al. 1991) but not previously for eggs of herring gull, Caspian tern (*Hydroprogne caspia*), or least tern (*Sternula antillarum*; Burger and Gochfeld 1995).

Molar ratios of Se to Hg differed substantially among species, with mean values for Audouin of 2.0 and for yellow-legged between 3.7 and 13.0. The mean molar ratios for both species were all greater than 1:1 (Fig. 4), with the lowest ratio value corresponding to Audouin gull in the Ebro Delta; for the yellow-legged gull the values decreased in the following order: Columbretes > Ebro Delta > Dragonera > Mar Menor > Cies > Berlengas > Medes > Chafarinas. Eggs with higher levels of Hg generally had lower molar ratios (Fig. 4), due to high Se concentrations. In fact, in sampling sites close to agricultural activities (such as the Ebro Delta), Se levels are high due to its considerable use in agriculture, as an additive to insecticides, fertilizers, and foliar sprays (Kabata-Pendias 2011).

## Conclusions

Mercury concentrations in yellow-legged gull eggs from the Iberian Peninsula appeared to have remained stable between 2009 and 2016 but tended to be higher in the NM (Ebro Delta and Columbretes) than in the SM or the Atlantic colonies. This spatial variation is likely to be the result of a combination of local pollution, oceanic currents, and birds feeding habits. Independently, concentrations of Hg and Se in the yellow-legged were below those associated with embryotoxicity, but there was a positive correlation between Hg and Se concentrations and these two elements can act synergistically to exert embryotoxicity.

Hg concentrations in eggs of Audouin’s gulls from the Ebro Delta were three to four times higher than those of yellow-legged gulls from the same area. This may be the result of this species taking more fish than yellow-legged gulls do, including demersal fish discards from fishing boats. The Hg concentrations in Audouin’s gull eggs are of a magnitude associated with embryotoxicity in other species. Mercury levels in Audouin’s gull are more than ten times above the benchmark suggested to reduce nest success by 10%. Further investigation of the scale and effects of Hg and Se contamination in Audouin’s gull eggs from the Ebro Delta is merited.
